# Mouse Sirt3 promotes autophagy in AngII-induced myocardial hypertrophy through the deacetylation of FoxO1

**DOI:** 10.18632/oncotarget.13429

**Published:** 2016-11-17

**Authors:** Jingyuan Li, Tongshuai Chen, Ming Xiao, Na Li, Shujian Wang, Hongyan Su, Xiaobin Guo, Hui Liu, Fangying Yan, Yi Yang, Yun Zhang, Peili Bu

**Affiliations:** ^1^ The Key Laboratory of Cardiovascular Remodeling and Function Research, Chinese Ministry of Education and Chinese Ministry of Health, The State and Shandong Province Joint Key Laboratory of Translational Cardiovascular Medicine, Qilu Hospital of Shandong University, Jinan, Shandong, China

**Keywords:** Sirt3, FoxO1, autophagy, myocardial hypertrophy, deacetylation modification

## Abstract

Sirt3, a mitochondrial NAD+-dependent histone deacetylase, is the only member proven to promote longevity in mammalian Sirtuin family. The processed short form of Sirt3 has been demonstrated to target many mediators of energy metabolism and mitochondrial stress adaptive program. Autophagy serves as a dynamic recycling mechanism and provides energy or metabolic substrates. Among the mechanisms triggered by cardiac stress, opinions vary as to whether autophagy is a protective or detrimental response. Here, by inducing the Sirt3-knockout mice to myocardial hypertrophy with chronic angiotensin II infusion for four weeks, we determined the role of Sirt3 in myocardial hypertrophy and autophagy. In this study, the Sirt3-knockout mice developed deteriorated cardiac function and impaired autophagy compared to wild-type mice. What's more, the overexpression of Sirt3 by lentivirus transfection attenuated cardiomyocytes hypertrophy by promoting autophagy. We further demonstrated that Sirt3 could bind to FoxO1 and activate its deacetylation. Sequentially, deacetylated FoxO1 translocates to the nucleus where it facilitates downstream E3 ubiquitin ligases such as Muscle RING Finger 1 (MuRF1) and muscle atrophy F-box (MAFbx, Atrogin1). Altogether, these results revealed that Sirt3 activation is essential to improve autophagy flux by reducing the acetylation modification on FoxO1, which in turn alleviates myocardial hypertrophy.

## INTRODUCTION

Myocardial hypertrophy is an important adaptive response to increased hemodynamic stress, allowing the organism to maintain or increase its cardiac output during the early period [1–3]. However, sustained stressors would burden the hypertrophic cardiomyocytes, ultimately causing congestive heart failure or even sudden death due to arrhythmias [4, 5]. Massive evidence has demonstrated the mechanisms of myocardial hypertrophy at multiple levels, such as alterations of molecular signalling pathways, adverse changes in subcellular organelles and communications among various cell types in the heart [6–9].

Of these molecular mechanisms involved in myocardial hypertrophy, increasing evidence showed that autophagy is essential to maintain cardiac function and cellular homoeostasis. Autophagy is well known as a conserved cellular catabolic pathway that eliminates defective proteins or organelles and removes intracellular pathogens [10, 11]. Under normal circumstances, it maintains at a low basal level for intracellular homoeostasis [12] and component renovation [13]. In specific contexts, up-regulated autophagy can be an adaptive process and protects against pathological changes of many diseases [11]. A large body of data demonstrated that one of the prominent features of myocardial hypertrophy is autophagy dysfunction [14, 15]. Thus, understanding such mechanisms of autophagic regulation may facilitate a novel and useful strategy for management of myocardial hypertrophy without provoking heart failure.

Recent studies reported that Sirtuins play critical roles in regulating cellular processes, inhibiting metabolic disorders and combating associated diseases [16–18]. Sirtuins belong to the family of NAD+-dependent class III histone deacetylases. Sirt3 is the only member among the seven Sirtuins (Sirt1-7) that relates to longevity in humans [19]. It can act on both the nuclear H3, H4, Ku70 [20] and the mitochondrial acetyl-CoA-synthetase2, long-chain acetyl-CoA dehydrogenase [21]. Though Sirt3-deficient mice didn't show visible phenotypes, they developed evident myocardial hypertrophy and interstitial fibrosis at eight weeks of age [22, 23]. Sirt3 can block cardiac hypertrophic response by reducing ROS production [22, 24, 25]. Nevertheless, whether there are other regulatory signalling pathways for Sirt3 to alleviate myocardial hypertrophy remains a mystery.

The Forkhead boxO transcription factors (FoxOs) are conserved proteins that regulate multiple signalling pathways important for stress resistance, metabolism, cell cycle arrest and apoptosis [26]. A recent series of studies have indicated that FoxO1 acetylation is pivotal in regulating the expression of autophagy genes, which can be mediated by dissociation from Sirt2 and histone deacetylase inhibitors [27–31]. However, whether Sirt3 could regulate the activity of FoxO1 during autophagy remains to be explored.

In the current study, we investigated the role and specific mechanism of Sirt3 in myocardial hypertrophy and autophagy. To this end, our results showed that Sirt3-FoxO1 signalling pathway is in charge of enhancing autophagy, which can serve as a pro-survival mechanism in angiotensin II (AngII)-mediated myocardial hypertrophy.

## RESULTS

### Sirt3 deficiency aggravates AngII-induced murine myocardial hypertrophy

**Figure 1:**
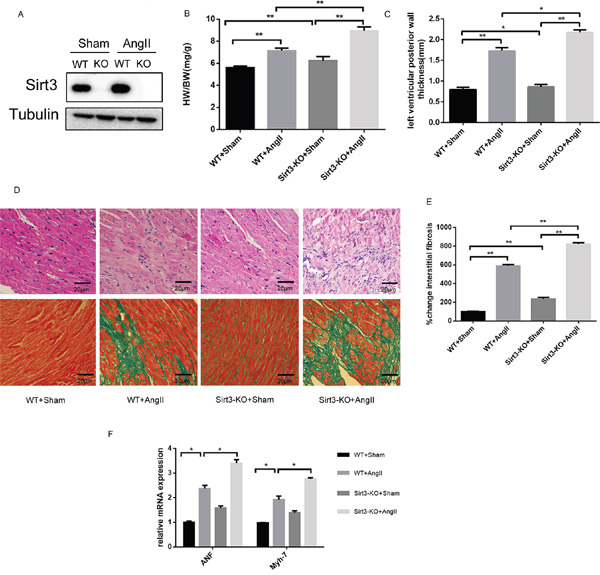
Sirt3 deficiency aggrevates AngII-induced murine myocardial hypertrophy **A**. Immunoblot analysis of the short form of the Sirt3 was performed in sham and AngII-treated WT and Sirt3-KO mice hearts. Tubulin expression was used as loading control. **B**. Ratio of the heart weight to body weight in WT and Sirt3-KO mice infused with either saline or AngII for 4 weeks. (n=5) **C**. The left ventricular wall thickness was measured with echocardiology as described in the methods section. (n=5) **D-E**. Hematoxylin/eosin stained cardiac sections from control or AngII-treated WT and Sirt3-KO mice showed cardiomyocyte loss or dropout. Masson's trichrome stained sections of the hearts were to detect fibrosis (blue). The graph showed the quantification of interstitial fibrosis in sham or AngII-treated WT and Sirt3-KO mice. (n=5) Scale bar: 20 μm. **F**. ANF and Myh7 mRNA levels in heart samples of sham or AngII-treated WT and Sirt3-KO mice. (n=5) The data are presented as the means ± SEM of three independent experiments. *P<0.05, **P<0.01.

To validate whether Sirt3 during myocardial hypertrophy was protective, we subjected both the WT and Sirt3-KO mice to chronic AngII infusion for four weeks. Firstly, immunoblot analysis confirmed there was no Sirt3 protein in the Sirt3-KO murine hearts. Furthermore, we observed increased Sirt3 expression in the hypertrophic hearts of mice subjected to AngII (Figure 1A). We also found the heart weight/body weight (HW/BW) and left ventricular posterior wall thickness in Sirt3-KO mice were higher compared to WT mice at baseline and increased further after chronic infusion (Figure 1B-1C). Also the histological staining results showed an obvious increased interstitial fibrosis in both AngII-treated Sirt3-KO mice and their WT controls (Figure 1D-1E). The transcription activities of hypertrophic markers, atrial natriuretic peptide (ANP) and myosin, heavy chain 7, cardiac muscle, β (Myh7) revealed a consistent trend with histological staining. Altogether, these results indicated that Sirt3 might be involved in preventing myocardial hypertrophy.

### Sirt3 regulates autophagy flux in vivo

**Figure 2:**
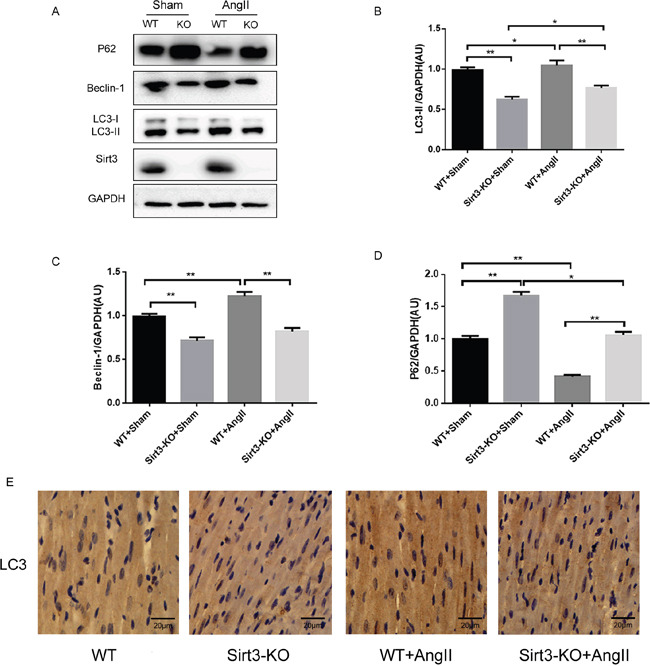
Sirt3 regulates autophagy flux in vivo **A-D**. Immunoblot analysis of LC3, Beclin-1 and p62 in sham and AngII-treated WT and Sirt3-KO murine hearts. GAPDH expression was used as loading control. Bar graphs showed the quantification of LC3-II, Beclin-1 and p62 measured by densitometry analysis. (n=5) **E**. Immunohistochemical analysis of autophagic marker LC3. Scale bar: 20 μm. The data are presented as the means ± SEM of three independent experiments.*P<0.05, **P<0.01.

Autophagy is a highly conserved protein degradation mechanism which involves removal and recycling of damaged proteins and organelles, and consequently supports cell survival under stress. To investigate the protective mechanism of Sirt3 in hypertrophy, we analysed autophagic flux in vivo by Western blot. The results suggested that Sirt3 knockout caused a significant reduction in the expression of LC3-II and Beclin-1, while an increase in p62, a link between LC3 and ubiquitinated substrates, revealed a reduction in autophagic degradation (Figure 2A-2D). A similar autophagy-inhibiting effect was observed in the Sirt3-KO mice from immunohistochemistry assay (Figure 2E). Taken together, these data indicated that Sirt3 attenuated AngII-induced myocardial hypertrophy by promoting the autophagic process.

### Sirt3 activation by AngII increases autophagy flux in vitro

**Figure 3:**
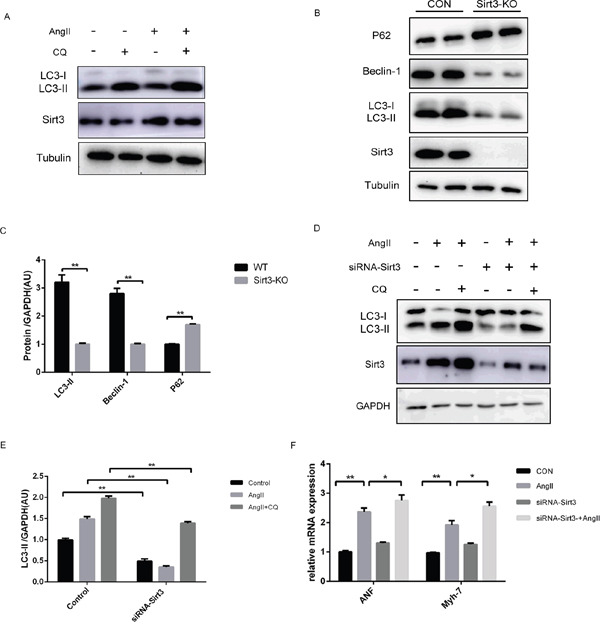
Sirt3 activation by AngII increases autophagy flux in vitro **A**. Immunoblot analysis of Sirt3, LC3 was performed on primary neonatal rat cardioyocytes treated with AngII (1μM, 24h) or chloroquine (CQ, 60μM, 16h). Tubulin expression was used as loading control. **B-C**. Immunoblot analysis of Sirt3 and autophagic markers was performed on primary neonatal cardioyocytes from WT and Sirt3-KO mice. Tubulin expression was used as loading control. Bar graphs showed the quantification of LC3-II, Beclin-1 and p62 measured by densitometry analysis. (n=5) **D-E**. The H9C2 cardiomyocytes were transfected with siRNA-Sirt3, and then treated with CQ and AngII. GAPDH expression was used as loading control. Bar graph represents quantification of LC3-II levels measured by densitometry analysis. (n=5) **F**. The bar graph showing the quantification of ANF and Myh7 mRNA levels as in D. (n=5) The data are presented as the means ± SEM of three independent experiments.*P<0.05, **P<0.01.

For further confirmation of the mechanisms under hypertrophy, we treated primary neonatal cardiomyocytes with AngII and found that both Sirt3 and LC3-II expression were elevated (Figure 3A). Increased LC3-II levels can signify either an increase of autophagy initiation, a block of downstream lysosomal degradation, or both. Chloroquine (CQ), a specific compound that neutralizes the lysosomal pH, prevents autophagy by inhibiting lysosomal degradation. A higher level of LC3-II in AngII+CQ group compared to CQ alone may indicate that Sirt3 promoted the synthesis of autophagy-related membranes and accumulation of autophagosomes. In addition, we isolated primary neonatal cardiomyocytes from WT and Sirt3-KO mice. Immunoblot analysis indicated that the LC3-II and Beclin-1 expression were decreased in the Sirt3-KO group, while the p62 level was elevated (Figure 3B-3C). This was consistent with results in vivo and revealed that Sirt3 deficiency also inhibited lysosomal degradation.

Furthermore, we treated the cardiomyocytes H9C2 cell line with AngII or CQ. The results were consistent with that observed in primary neonatal cardiomyocytes (Supplementary Figure 1A-1C). With Sirt3 levels evidently decreased by siRNA transfection in H9C2, the reduced LC3-II was also observed in the AngII+CQ group, indicating lower autophagosome accumulation (Figure 3D-3E). Additionally, the siRNA-Sirt3 group showed a higher level of transcription activities of hypertrophic markers, compared to its corresponding controls (Figure 3F).

### Sirt3 overexpression triggers autophagy in H9C2 cells

**Figure 4:**
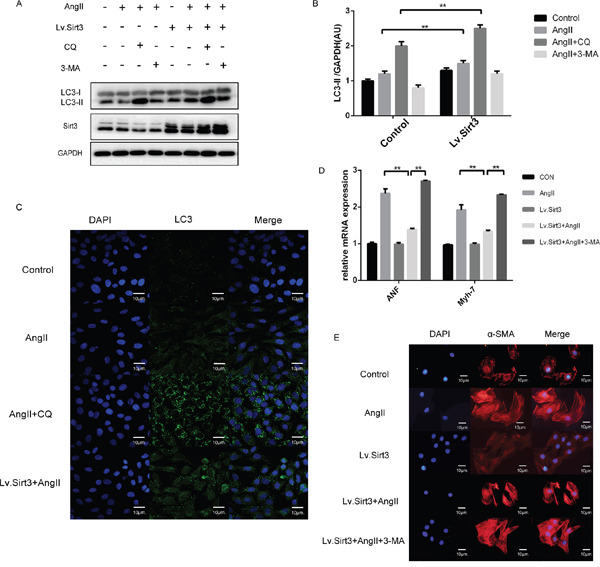
Sirt3 overexpression triggers autophagy in H9C2 cells **A-B**. Immunoblot analysis of Sirt3, LC3 was performed on H9C2 extracts with or not lentivirus infection (MOI=10). The cardiomyocytes was treated by lentivirus infection and then stimulated with CQ and 3-MA (5mM, 8h) before the end of AngII. GAPDH expression was used as loading control. Bar graphs showed the quantification of LC3-II measured by densitometry analysis. (n=5) **C**. Immunofluroscence analysis of LC3 in H9C2 pretreated with or not CQ before the end of AngII stimulation. DAPI stained nucleus in blue. Images were from digital confocal microscopy core facility. Scale bar: 10 μm. **D**. The bar graph showing the quantification of ANF and β-MHC mRNA levels. (n=5) **E**. Immunofluroscence analysis of α-SMA in H9C2 with or not lentivirus infection (MOI=10). DAPI stained nucleus in blue. Images were from fluorescence microscopy. Scale bar: 10 μm. The data are presented as the means ± SEM of three independent experiments.*P<0.05, **P<0.01.

To obtain additional evidence for the effect of Sirt3 on autophagy, we infected H9C2 cell line with lentivirus (Lv) for Sirt3 at an MOI of 10 for 24h. With Sirt3 highly expressed, LC3-II levels increased in AngII+CQ group (Figure 4A-4B). Sirt3-induced autophagosome formation was also confirmed by fluorescence imaging (Figure 4C). Here to text whether the increased autophagy induced by Sirt3 overexpression played a beneficial or and detrimental role in the heart, we interfered the autophagy flux with 3-methyladenine (3-MA). 3-MA, as an inhibitor of class III phosphoinositide-3-Kinase, can inhibit the autophagosome formation. LC3-II levels decreased after 3-MA stimulation in both control and Lv.Sirt3-infected cells, compared with their corresponding control groups (Figure 4A). In contrast, the transcription activities of hypertrophic markers and immunofluorescence analysis of α-SMA indicating that impaired autophagy potentiated cardiomyocyte hypertrophy (Figure 4D-4E).

In summary, our results demonstrated the importance of Sirt3 in promoting autophagy and supported that autophagy at this level can act as a protective mechanism to counteract the hypertrophic effect of AngII.

### Sirt3-regulated acetylation status of FoxO1 is involved in autophagy

**Figure 5:**
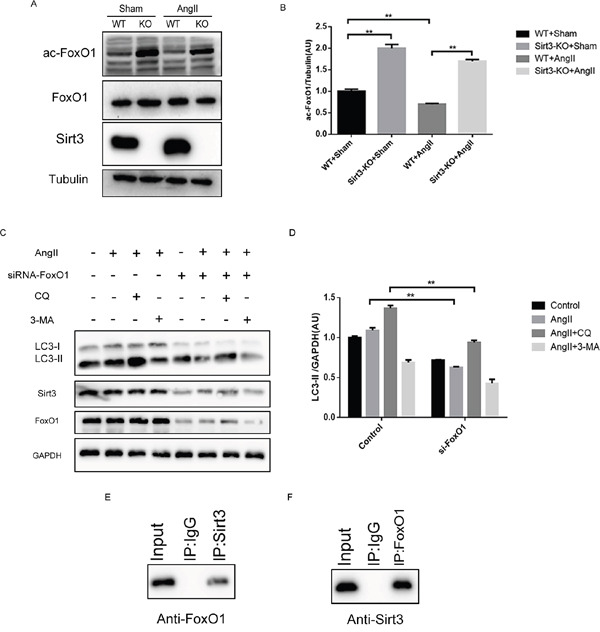
Sirt3 controls the acetylation status of FoxO1 **A-B**. Immunoblot analysis of FoxO1 and ac-FoxO1 was performed in sham and AngII-treated WT and Sirt3-KO murine hearts. Tubulin expression was used as loading control. Bar graph represents quantification of ac-FoxO1 levels measured by densitometry analysis. (n=5) **C-D**. Immunoblot analysis of autophagy biomarkers and Sirt3 in the control and siRNA-FoxO1 groups. GAPDH expression was used as loading control. Bar graph represents quantification of LC3-II levels measured by densitometry analysis. (n=5) **E**. Cellular extracts were immunoprecipitated with Sirt3 antibody and analyzed with anti-FoxO1. **F**. Cellular extracts were immunoprecipitated with FoxO1 antibody and analyzed with anti-Sirt3. The data are presented as the means ± SEM of three independent experiments.*P<0.05, **P<0.01.

Though these results demonstrated that Sirt3 could regulate autophagy process, the underlying mechanism remains unclear. FoxO1 is regarded as an important mediator of hypertrophy for its ability to activate multiple downstream gene sets. What's important is that the acetylation level of FoxO1 is obviously higher in the Sirt3-KO mice than that in WT mice (Figure 5A-5B). We proposed the hypothesis that FoxO1 deacetylation is involved in the Sirt3-mediated autophagic signalling pathways. Firstly, we treated the cardiomyocytes with siRNA-FoxO1 and AngII. The data showed that FoxO1 silencing indeed blocked the autophagic induction (Figure 5C-5D). The cellular proteins were immunoprecipitated and analyzed by Western blot antibodies specific to FoxO1 or Sirt3. Thus it can be concluded that there exists interaction between Sirt3 and FoxO1 (Figure 5E-5F).

**Figure 6:**
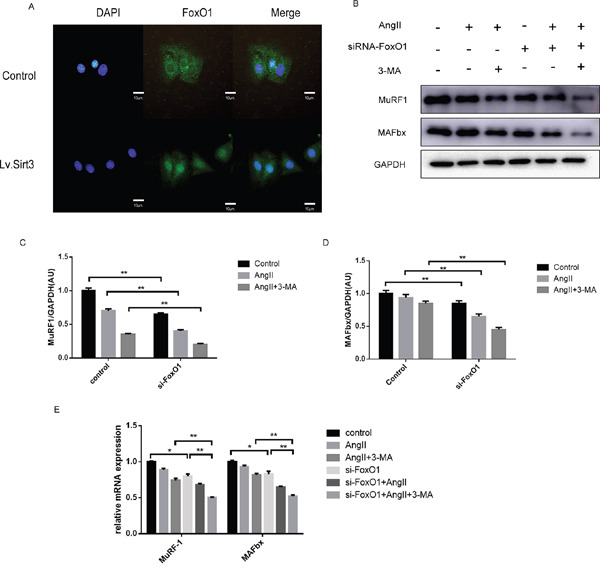
Sirt3 promotes the nuclear translocation and transcriptional activity of FoxO1 **A**. Immunofluroscence analysis of FoxO1 in H9C2 with or not lentivirus infection (MOI=10). DAPI stained nucleus in blue. Images were from fluorescence microscopy. Scale bar: 10 μm. **B-D**. Immunoblot analysis of MuRF1 and MAFBx in the control and siRNA-FoxO1 groups. GAPDH expression was used as loading control. Bar graph represents quantification of MuRF1 and MAFBx levels measured by densitometry analysis. (n=5) **E**. The bar graph showing the quantification of MuRF1 and MAFBx mRNA levels. (n=5) The data are presented as the means ± SEM of three independent experiments.*P<0.05, **P<0.01.

We adopted the imunofluorescence assay to investigate the localization of FoxO1. Results showed that with Sirt3 highly expressed, more cytoplasmic FoxO1 was translocated to the nucleus (Figure 6A). One effect of FoxO1 is related to E3 ubiquitin ligases such as Muscle RING Finger 1 (MuRF1) and muscle atrophy F-box (MAFbx), which are rather important for regulating muscle mass. We found that the mRNA expression and protein level of MuRF1 and MAFbx were reduced in the si-FoxO1 group or AngII group (Figure 6B-6E).

These results together indicated that Sirt3 might be able to deacetylate FoxO1 so as to promote its nuclear translocation and transcriptional activity, which is one important mechanism underlying Sirt3-mediated autophagic process. What's more, we found that with the knockdown of FoxO1, the expression of Sirt3 also downregulate at a considerable extent. We speculate there exists positive feedback between Sirt3 and FoxO1, that is Sirt3 promotes FoxO1 nuclear translocation and nuclear FoxO1 acts as a transcription factor to bind to the Sirt3 gene and to promote its transcription.

## DISCUSSION

The present study elucidates the critical role of Sirt3 in autophagy during pathological myocardial hypertrophy. Using in vivo and in-vitro hypertrophy models, we demonstrated for the first time that the knockdown of Sirt3 suppressed autophagy and Sirt3-FoxO1 signalling pathway mediated the autophagy flux. Sirt3 was compensatorily increased under AngII stimulation in the murine hearts and primary cardiomyocytes. Its activation alleviated myocardial hypertrophy by deacetylating FoxO1 and inducing its nuclear translocation, which in turn promoted cellular autophagy.

Post-translational modifications, including phosphorylation, ubiquitination and acetylation, are crucial for the regulation of eukaryotic protein activities. The role of acetylation in autophagy control has only recently emerged to research attention [32]. The mechanism as to how the protein acetylation modulates autophagy, however, remains controversial. For one thing, deacetylation of autophagy-associated proteins, such as ATG5, ATG7, and ATG12, is involved in autophagy [32, 33]. Moreover, autophagy can be stimulated by deacetylases activation or acetyltransferases inhibition [34, 35]. For another, some evidence showed that hyperacetylation of ATGs, such as Atg3 in yeast [36] and ULK1 in human cells [37], is also implicated in starvation-induced autophagy. Here in the current study, we provided evidence that Sirt3 knockout, which leads to increased FoxO1 acetylation and reduced transcriptional activity, blocks autophagy flux. Our results don't reject the involvement of other HDACs-dependent FoxO1 deacetylations in the modulation of autophagy flux. Deacetylation of nuclear FoxO1 by Sirt1 could induce autophagy, which in this manner appears to be pro-survival [38]. Conversely, dissociation of FoxO1 from Sirt2 results in the hyperacetylation of FoxO1 which promotes autophagy and leads to cell death [28, 39]. Although the three of Sirtuins all belong to class III histone deacetylases family, the function and underlying signalling pathways differ greatly. This phenomenon might be associated with the Sirtuins’ varied subcellular localization. Sufficient literature reported that Sirt1 is found in the nucleus, Sirt2 is primarily cytosolic and Sirt3 exists in both mitochondria and nucleus [40]. It is assumed that the exact effect of protein deacetylation on autophagy regulation might be context-dependent and molecular-specific.

The conserved autophagic-lysosomal pathway is necessary to degrade damaged organelles and sustain normal cardiac function. However, a consensus is yet to be reached regarding whether autophagy is a compensatorily protective mechanism induced by various stresses. Many studies proved that baseline and upregulation of autophagy maintain intracellular hemostasis and protect from myocardial hypertrophy. Cardiac-specific atg5-deficient and Gsk3α-knockdown mice both showed inhibited autophagy accompanied with aggravated hypertrophy and reduced cardiac contractility [41, 42]. On the contrary, upregulation of autophagy has been found to be closely associated with myopathy. Pathological myocardial remodelling induced by severe pressure showed a significantly increased Beclin-1 and can be blocked by disrupting the gene encoding Beclin-1 [43]. In spite of controversial conclusions about the autophagy effect on hypertrophy, autophagy, in fact, has dual functions and whether it acts as an adaptive or maladaptive role depends on the degree of autophagy activation, stage and severity of different diseases [44]. In our experimental condition, Sirt3-KO mice, both sham and AngII-treated groups, exhibited greater extents of HW/PW, LV posterior wall thickness and interstitial fibrosis ratio compared with WT mice. Cases of serious hypertrophy were accompanied with a lower level of autophagic biomarkers LC3-II and Beclin-1. The results in vitro revealed that enhanced autophagy by Sirt3 overexpression alleviated AngII-induced cardiomyocytes hypertrophy. Considering these findings, we put forward that the autophagy activation plays a more cardioprotective than detrimental role to maintain structural and functional homoeostasis in this particular context.

Primary cardiomyocytes usually are preferred models to investigate the cellular and molecular changes of hearts, which involves cell size increase, contractile proteins reorganization, genes regulation and so on. However, these studies require lots of animals [45]. It is worth noting that H9C2 cell line originates from embryonic rat ventricular tissue, while hypertension-associated cardiac hypertrophy is most significant in the ventricular muscle [45]. In addition, this cell is similar to primary cardiomyocytes in many aspects, such as membrane morphology and electrophysiological properties [46]. Above all, H9C2 cells present a good deal of hypertrophic traits under AngII, which are similar to those in primary cardiomyocytes [45]. We also found that the tendency of LC3-II and p62 in primary neonatal cardiomyocyte and H9C2 were consistent. Therefore we think H9C2 can act as a reasonable model in vitro to investigate autophagy under cardiomyocyte hypertrophy.

In conclusion, our data shed new lights on the intrinsic connection among Sirt3-FoxO1, cardiac hypertrophy and autophagy. Deacetylation of FoxO1 by Sirt3 promotes autophagy and alleviates myocardial hypertrophy. Besides, we found that knockdown of FoxO1 induced low Sirt3 expression level. There may exist a positive feedback between Sirt3 and FoxO1. Further interpretation of the interaction between Sirt3 and FoxO1 will deepen the comprehension of autophagy regulation and provide support for targeting autophagy as a new therapy for myocardial hypertrophy.

## MATERIALS AND METHODS

### Ethics statement

The animal experimental protocol complied with the Animal Management Rules of the Chinese Ministry of Health (Document No. 55, 2001) and was approved by Animal Care and Use Committee of Shandong University. Male global Sirt3 KO (129-SIRT3tm1.1Fwa/J) mice were obtained from Jackson Laboratories (Bar Harbor, ME) and their respective wild-type (WT) control (129S1/SvImJ) mice were purchased from Department of Laboratory Animal Science of Peking University as control (Beijing, China). The adult male mice (8 weeks old) were used in the study. All animals were fed with laboratory standard chow and water, and housed in individually ventilated cages at the key Laboratory of Cardiovascular Remodeling and Function Research in Qilu Hospital of Shandong University.

### Animal model

Mice were anesthetized with 2% isoflurane in oxygen, and an osmotic mini-pump (Alzet micro-osmotic pump model 1007D; Alzet DURECT Corporation, Cupertino, CA, USA) was implanted subcutaneously between the scapulae. Pumps were filled with AngII in sterile saline and were set to deliver AngII (200 ng/kg per/min). Control mice received infusion of saline of comparable volume. WT and Sirt3-KO mice were randomly assigned to the control group or Ang II-treated group. Animals were sacrificed four weeks after surgery and their hearts were removed to be analyzed for the development of myocardial hypertrophy and autophagy flux. Mini-pumps were weighed afterwards in order to verify complete diffusion.

### Reagents

Angiotensin II, chloroquine (CQ), Bafilomycin A1(Baf A1) and 3-methyladenine (3-MA) were purchased from Sigma Aldrich (Sigma Aldrich, USA). Primary antibodies for detecting Sirt3 (rabbit monoclonal) (D22A3) [5490], FoxO1 (rabbit monoclonal) (C29H4) [2880], LC3 (rabbit polyclonal) [2775], LC3 (rabbit polyclonal) [3868], Beclin-1 (rabbit monoclonal) (D40C5) [3495] were purchased from Cell signalling Technology (CST, UK). Primary antibodies against ac-FoxO1 (rabbit polyclonal) (FKHR D19) [sc49437], MuRF1 (mouse monoclonal) [sc398608], MAFBx (mouse monoclonal) (sc166806) were purchased from Santa Cruz Biotechnology (Santa Cruz, USA). Primary antibodies against p62 (mouse monoclonal) [ab56416] was obtained from Abcam. Primary antibodies against β-Tubulin (mouse monoclonal) [BM1453], GAPDH (mouse monoclonal) [BM1623], α-SMA (mouse monoclonal) [BM0002] was purchased from Boster.

### Echocardiograhy of mice

Echocardiography was carried out on lightly anesthetized (1% isoflurane in air) mice placed on a heating pad. Limb leads were attached for electrocardiogram gating. The ultrasound examination was achieved by a Visual Sonics Vevo 770 machine and a 30-MHz high-frequency transducer. We measured the diastolic and systolic function with M-mode, two-dimensional (2-D), pulse wave (PW) Doppler and tissue Doppler imaging (TDI). The operator was blind to the genotype of the mice.

### Isolation, culture of rat cardiomyocytes, transfection/infection

Primary cultures of cardiac myocytes were prepared from 2-day-old neonatal rat hearts as described previously [20]. H9C2 cell line was obtained from American Type Culture Collection (ATCC). Myocytes were maintained in Dulbecco's modified Eagle's medium (Gibco) supplemented with 10% fetal bovine serum and 1:100 penicillin/streptomycin (Invitrogen, Carlsbad, CA, USA) in a humidified incubator with an atmosphere of 5% CO2 at 37°C. The lentivirus of control and Sirt3 was purchased from Shanghai Genechem Co., LTD. For all lentivirus experiments, the virus was used at a multiplicity of infection (MOI) of 10. For the RNA interference experiments, the cardiomyocytes were transfected with 50 nM of small interfering RNA (siRNA) specific for Sirt3 or FoxO1 with Lipofectamine 2000 (Invitrogen) for 24h. The sequence of siRNA-Sirt3 is (5’-3’) GCGUUGUGAAACCUGACAUTTAU GUCAGGUUUCACAACGCTT. The sequence of siRNA-FoxO1 is (5’-3’) GAGGAUUGAACCAGUA UAATTUUAUACUGGUUCAAUCCUCTT.

### Histology and immunohistochemistry

The isolated hearts were fixed in 4% paraformaldehyde solution and embedded in paraffin. The samples were cut into 5-μm sections and stained with hematoxylin/eosin following standard procedure. Masson's trichrome staining were performed to evaluate myocardial fibrosis. Collagen volume fraction was quantified blindly by an automated image analysis system (Image-Pro Plus, Version 7.0, Media Cybernetics, Silver Spring, MD, USA). Cardiac fibrotic fraction was measured as a ratio of total interstitial collagen area to total area of the section while excluding perivascular collagen. Immunohistochemical analysis involved the antibody against LC3 (1:100 dilution). Measurements from 3 heart sections (8–10 fields per section) per rat were averaged for all parameters.

### Co-immunoprecipation

Co-immunoprecipitations were made on raft complexes, using 1μg of Sirt3 or FoxO1 monoclonal antibody covalently cross-linked on pan-rabbit IgG Dynabeads. Purified rabbit IgG was used for some of the negative controls. Antigen capture was performed by overnight incubation of IgG-loaded beads with a raft sample corresponding to protein diluted in 0.2ml immunoprecipitation buffer (10 mM Tris-HCl, pH 7.4, 150 mM NaCl, 1% Triton X-100). Magnetic beads carrying the immune complexes were subsequently washed five times in washing buffer (10 mM Tris-HCl, pH 7.4, NaCl 150 mM, 1% Triton X-100, 60 mM octyl β-D-glucopyranoside). Precipitated proteins were eluted by boiling the beads for 5 min in RIPA buffer (150 mM NaCl, 25 mM Tris-HCl, pH 7.5, 5 mM EDTA, 0.5% sodium deoxycholate, 0.5% NP 40, 0.1% sodium dodecyl sulfate [SDS]). Samples were subjected to immunoblotting using a primary antibody against FoxO1 or Sirt3 and a secondary antibody that recognized only native IgG.

### Western blot analysis

50-μg samples of the cell lysates were electrophoresed on 10% SDS/PAGE gels and the separated proteins were transferred onto PVDF membranes in an indicated time, blocked with 5% nonfat milk in TBS for 2h. The blocked membranes were separately incubated with the specific antibodies overnight at 4°C and washed three times with TBS buffer containing 0.1% Tween, 10 minutes each. The samples were then incubated in the secondary antibody conjugated to horseradish peroxidase (1:5000 dilutions with 1% nonfat milk in TBS) against to the primary antibody, and washed as steps described above at room temperature on a mild shaker. The protein bands were visualized through enhanced chemiluminescence (Millipore) and the protein levels were detected using an Image Quant LAS4000 chemiluminescence reader (GE, USA). Relative protein levels were quantified by using Image J software.

### Immunofluorescence analysis

The cardiomyocytes were fixed for 20min in cold methanol and then treated with 0.5% Triton X–100. The cells were incubated with primary antibodies for LC3 or FoxO1 (1:200, diluted in 1%BSA) overnight at 4°C in a humidified chamber followed by BSA blocking for 30 min and incubation in secondary antibodies for 1 h. All imaging analyses were performed in the digital confocal microscopy core facility or fluorescence microscopy.

### Real-time PCR analysis

Total RNA was extracted from cardiac left ventricles using TRIzol and 1μg samples of RNA were reverse-transcribed into cDNA using the Transcriptor First Strand cDNA synthesis kit. PCR amplifications were quantified using a MyiQ Real-Time PCR System (Bio-Rad) and GAPDH was used as the internal control. The primers for PCR analysis are as follows: ANP, forward 5’-TAAGCCCTTGTGGTGTGTCA-3’ and reverse 5’-GCAAGACCCC ACTAGACCAC-3’; β-MHC forward 5’-AAGGGCCTGAATGAGGAGTA-3’ and reverse 5’-AAAGGCTCCAGGTCTGAGG-3’; GAPDH forward 5’-CAAGATCATTGCTCCT CCTG-3’ and reverse 5’-TCATCGTACTCCTGCTTGCT-3’; MAFbx forward 5’-AGTAAGGCTGTTGGAGCTGAT-3’ and reverse 5’-GGACCAGCGTGCATAAGGAT-3’; MuRF1 forward 5’-CAGGGAACGACCGAGTTCAG-3’ and reverse 5’-GTGGCTGTTTTCCTTGGTCAC-3’.

### Statistical analysis

Statistical analyses were performed with SPSS21for Windows using one-way ANOVA and Tukey's HSD for post hoc analysis. A t-test was used for experiments with only two groups. A P-value<0.05 (two-tailed) was considered significant. Data are presented as the mean ± standard error of the mean.

## SUPPLEMENTARY FIGURE


