# Correction: Mouse Sirt3 promotes autophagy in AngII-induced myocardial hypertrophy through the deacetylation of FoxO1

**DOI:** 10.18632/oncotarget.28146

**Published:** 2022-05-24

**Authors:** Jingyuan Li, Tongshuai Chen, Ming Xiao, Na Li, Shujian Wang, Hongyan Su, Xiaobin Guo, Hui Liu, Fangying Yan, Yi Yang, Yun Zhang, Peili Bu

**Affiliations:** ^1^The Key Laboratory of Cardiovascular Remodeling and Function Research, Chinese Ministry of Education and Chinese Ministry of Health, The State and Shandong Province Joint Key Laboratory of Translational Cardiovascular Medicine, Qilu Hospital of Shandong University, Jinan, Shandong, China

**This article has been corrected:** In Figure 1D, the HE staining image of the ‘Sirt3-ko+sham’ group in Figure 1D (row 1, panel 3) is incorrect - the authors selected the wrong image from a group of similar files. The corrected Figure 1, produced using the original data, is shown below. The authors declare that these corrections do not change the results or conclusions of this paper.

Original article: Oncotarget. 2016; 7:86648–86659. 86648-86659. https://doi.org/10.18632/oncotarget.13429

**Figure 1:**
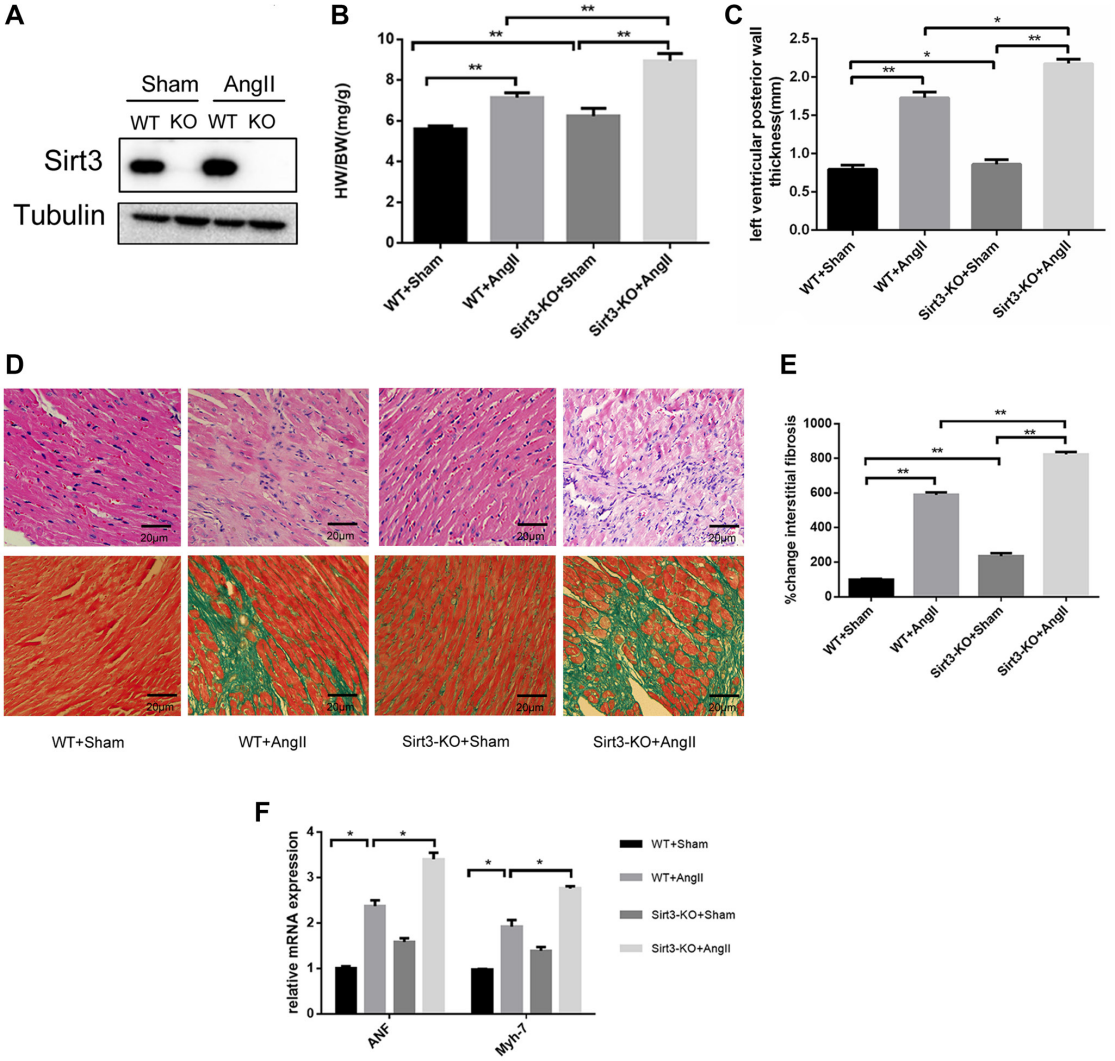
Sirt3 deficiency aggrevates AngII-induced murine myocardial hypertrophy. (**A**) Immunoblot analysis of the short form of the Sirt3 was performed in sham and AngII-treated WT and Sirt3-KO mice hearts. Tubulin expression was used as loading control. (**B**) Ratio of the heart weight to body weight in WT and Sirt3-KO mice infused with either saline or AngII for 4 weeks (*n* = 5). (**C**) The left ventricular wall thickness was measured with echocardiology as described in the methods section (*n* = 5). (**D**–**E**) Hematoxylin/eosin stained cardiac sections from control or AngII-treated WT and Sirt3-KO mice showed cardiomyocyte loss or dropout. Masson’s trichrome stained sections of the hearts were to detect fibrosis (blue). The graph showed the quantification of interstitial fibrosis in sham or AngII-treated WT and Sirt3-KO mice (*n* = 5). Scale bar: 20 μm. (**F**) ANF and Myh7 mRNA levels in heart samples of sham or AngII-treated WT and Sirt3-KO mice (*n* = 5). The data are presented as the means ± SEM of three independent experiments. ^*^ *P* < 0.05, ^**^ *P* < 0.01.

