# Supplementary material for: Mouse Sirt3 promotes autophagy in AngII-induced myocardial hypertrophy through the deacetylation of FoxO1
Source: Oncotarget. 2016 Nov 17;7(52):86648–59. doi: 10.18632/oncotarget.13429 (PMC5349942; doi:10.18632/oncotarget.13429)
Supplement: Supplementary file 1 [file oncotarget-07-86648-s001.pdf]

Mouse Sirt3 promotes autophagy in AngII-induced myocardial hypertrophy through the deacetylation of FoxO1

SUPPLEMENTARY FIGURE

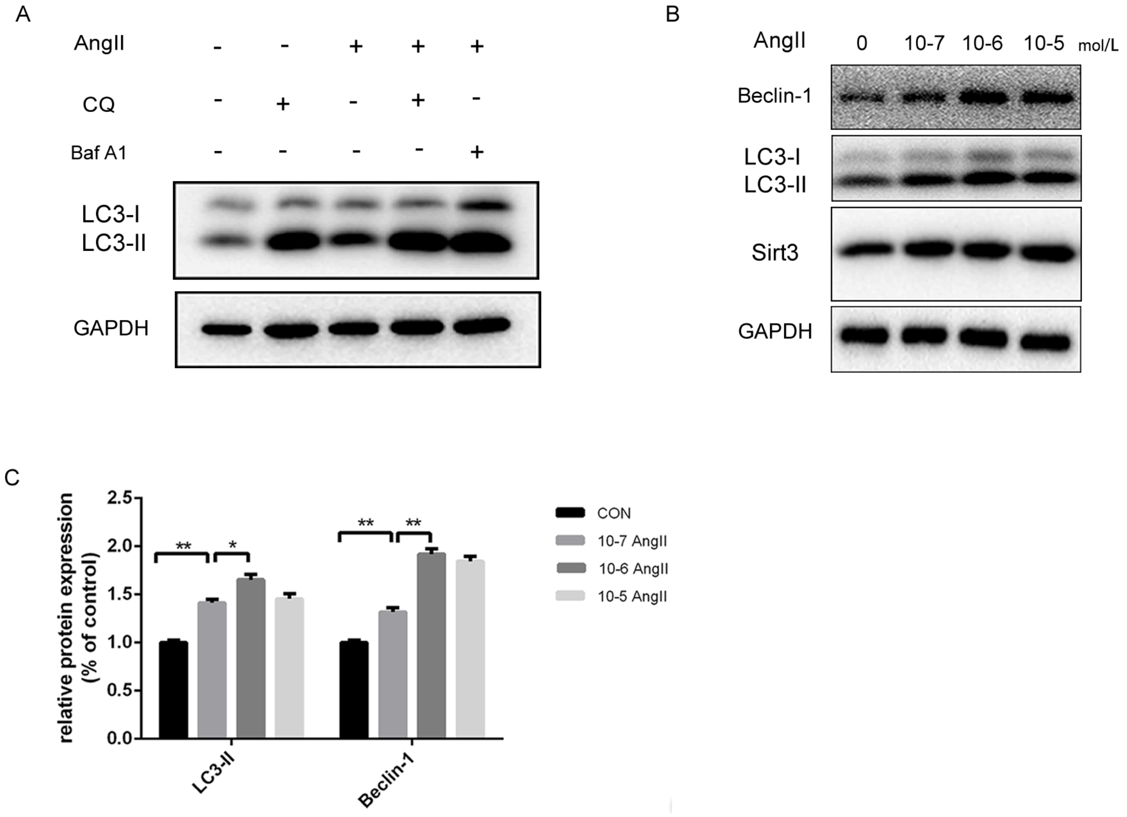

Supplementary Figure S1: Activation of Sirt3 by AngII treatment in H9C2 cell line promotes autophagy. A. Immunoblot analysis of Sirt3 and LC3 was performed on H9C2 extracts pretreated with or not chloroquine and Bafilomycin A1 (Baf1, 50 nM, 1 h) before the end of AngII stimulation. GAPDH expression was used as loading control. **B-C.** Immunoblot analysis of Sirt3, LC3 and Beclin-1 was performed on H9C2 extracts treated with AngII stimulation for different concentrations. GAPDH expression was used as loading control. Bar graph represents quantification of LC3-II and Beclin-1 levels measured by densitometry analysis. (n=5) The data are presented as the means ± SEM of three independent experiments.*P<0.05, **P<0.01.
